# On the nature of cancer and why anticancer vaccines don't work

**DOI:** 10.1186/1475-2867-5-25

**Published:** 2005-08-01

**Authors:** Richmond T Prehn

**Affiliations:** 1 Dept of Pathology, University of Washington, 5433 South Hudson Street, Seattle, WA 98118, USA

## Abstract

In this essay I suggest that the major difficulty in producing effective anti-cancer vaccines lies in the fact that most cancers have little immunogenicity because of a basic paucity of tumor-specific antigenicity. The lack of antigenicity, despite extensive genomic instability, could be explained if most tumor mutations occur in silenced genes. A further problem is that an immune reaction against tumor antigens, especially in moderate or low amount, may be stimulatory rather than inhibitory to tumor growth.

## 

It is now almost half a century since the overthrow of Ehrlich's doctrine of "horror autotoxicus" and the general acceptance of the contrary idea that animals can indeed be immunized against the growth of a transplanted syngeneic cancer. Why then is it that, despite nearly 50 years of intense investigation, attempts to use the immune reaction as a tool against cancer have, with the exception of bladder cancer, met with only moderate success? What is the realistic prospect that the next 50 years will see an improvement in this dismal state of affairs? Many investigators, myself included, have a large vested interest in the field of cancer immunology and will be reluctant to entertain any discouraging viewpoint, but the actual facts are, I believe, discouraging.

## Background

As a part of the original demonstration that syngeneic anticancer immunity is possible, it was shown that, among sarcomas induced in mice by a hydrocarbon, each tumor, when transplanted, could arouse an inhibitory immune reaction against itself [1]. However, it also became very clear that each tumor was antigenically unique, even if each had been induced by identical means in one and the same animal; although cross-reactions were reported, these were the exception [2,3]. Since it is probable that the immunogenicity was caused by the mutations induced by the mutagenic carcinogen, it was obvious that these chemicals produced a different spectrum of mutations in each tumor and with very little overlap. Consequently, one had to conclude that any of a vast array of possible mutations could be found in phenotypically similar cancers, a not impossible idea. However, it was also clear that none of the carcinogen-induced mutations, at least among those identified by their resulting antigenicity, could be considered essential or causative for the induction of the cancer.

Not surprisingly, although sarcomas induced by the same concentration of oncogen displayed a wide range of immunogenic strengths, there was found to be a positive statistical correlation between the strength of the immunogenicity and the concentration of the inducer [4,5]. Unfortunately, this relationship means that tumors induced by low concentrations of inducer, or that arise without obvious cause, tend to have either a very low or a nondetectable immunogenicity [6]. It is my hypothesis, as presented in this essay, that the paucity of immunogenicity reflects, for the most part, a basic lack of tumor-specific antigenicity rather than a blocking or suppression of an immune response.

## Immunosurveillance

It has been argued that the apparent general lack of tumor immunogenicity may be an artifact caused by immune selection for nonimmunogenic tumor variants. Perhaps most tumors, in accord with the immunosurveillance hypothesis, are really highly immunogenic and what we see is actually a small surviving, relatively non-immunogenic, highly selected subpopulation. This popular concept, judged by at least four types of evidence, is probably incorrect and thus cannot account for the paucity of tumor immunogenicity.

Firstly, immunological depression by any means usually has little if any facilitating effect on oncogenesis. Thus, chemical oncogenesis is not obviously altered by immunodepression [7]; the tumor immunogenicity and/or the degree of immune depression must be very carefully titrated in order to see any effect of immune depression, either positive or negative [8,9]. Both within and without the immunologically isolated environment of intraperitoneal diffusion chambers, nonimmunogenic tumors are commonly induced [10,11]. Furthermore, chemically-induced tumorigenesis may actually be lessened, not increased, in immunologically crippled, germ-free nude mice as compared with their essentially normal heterozygous nude controls [8].

Secondly, often a minute dosage of highly immunogenic transplanted cancer cells may grow when a somewhat larger inoculum fails [12]; this "sneaking through phenomenon", which can occur even in specifically immunized animals, again suggests that small incipient tumors could probably not be effectively surveyed.

Thirdly, even highly immunogenic hydrocarbon-induced sarcomas may fail to induce immunity if the tumor is left undisturbed *in situ*, so how could more ordinary tumors of lesser immunogenicity kindle a surveillance mechanism? In fact, to arouse an inhibitory immunity in the primary autochthonous host is difficult and, at least with hydrocarbon-induced sarcomas, requires repeated immunizations [13]. More often the challenge tumor, inoculated back into the animal in which it had originated, grew better than it did in other animals of the same strain, ie., better than it did in animals that had never before been exposed to that tumor [14,15]. This latter observation is best interpreted by the immunostimulation hypothesis [16] which I will now discuss.

## Immunostimulation

As compared with normal spleen cells, low proportions of spleen cells from immunized mice stimulated rather than inhibited the proliferation of admixed tumor cells when the mixture was injected subcutaneously. In this so called "Winn test" [17], larger proportions of immune spleen cells inhibited the growth of the same admixed tumor cells. As previously discussed, most tumors, arising from low concentrations of inducing agents, are expected to have little immunogenicity. The little immune response these tumors might engender would be expected, judging by these Winn test results, to stimulate rather than inhibit tumor growth. This was emphatically confirmed in a variety of extensive studies which showed, among other things, that a newly induced *in situ *mouse tumor, mesenchymal or epithelial, was stimulated to grow faster (had a shorter latency) if it could engender an immune response [8,11]. Even tumors that subsequently were shown to be highly immunogenic usually grew faster than tumors of little or no immunogenicity when the tumors were left undisturbed in their primary hosts [11]. Therefore, when immunodepression does appear to favor oncogenesis, this result, in many cases, is probably not because an immunological inhibitor to tumor growth has been reduced, but rather because the immune reaction has been depressed to a more stimulatory level [18]. Also, the possibility of doing harm by attempts at immunotherapy need to be carefully cosidered. For more extensive reviews and other evidences of tumor immunostimulation see [8,11,18,19].

The fact that a moderate tumor-specific immune reaction apparently favors the growth of an undisturbed *in situ *tumor, seems adequate to rule out any immunosurveillance of incipient cancers. Thus, the paucity of tumor immunogenicity is probably not caused by immunoselection. Rather, most types of tumor are apparently created *de novo *with little tumor-specific immunogenicity unless the tumors were induced by a large concentration of carcinogen and/or by an oncogenic virus. In fact, at least among hydrocarbon-induced mouse sarcomas, whatever little immune selection there may be apparently favors, rather than inhibits, the proliferation of a nacsent tumor.

## Role of Mutation

If cancer is based, in accord with the current paradigm, upon somatic mutations, why would most tumors have so little specific immunogenicity? While this question has many possible answers, it seems to me that, in the absence of immunoselection, there are two prime possibilities: either the somatic mutation idea of carcinogenesis is incorrect and/or the tumor mutations occur, for the most part, in silenced genes that are incapable of producing an antigenic product.

The hypothesis that the somatic-mutation paradigm is incorrect has been advanced over a number of years by a number of brave heretics [20-26]. They suggest that the mutations seen in neoplasia may not be causative; they are, instead, probably incidental or secondary. Primary epigenetic rather than genetic changes are postulated to result in the neoplastic phenotype. This hypothesis has the great virtue, as compared with the mutational paradigm, in that a tumor's reversion to a normal phenotype can be more easily understood. Reversion to a normal phenotype is indeed observed in a variety of laboratory experiments as well as in some clinical settings [26,27]. The real cause of most cancers is postulated by the heretics to reside in disrupted cell to cell signaling or some other epigenetic alteration which changes the spectrum of gene expression to produce a neoplastic phenotype. The many cogent arguments put forth by the heretics, entirely apart from any considerations of immunogenicity, have been well reviewed elsewhere [20-26]. However, these ideas of an epigenetic etiology fit well with the facts concerning the general lack of immunogenicity of cancers and could help to explain why immunity has not as yet lived up to its expected diva role in cancer therapy and prevention.

My personal view of the intimate details of carcinogenesis incorporates two suppositions, for each of which there is supporting experimental evidence. I will therefore, for purposes of this discussion, consider both of the following statements to be correct. The first is that DNA repair is defective or nonexistent in untranscribed or silenced genes [28]. Secondly, DNA mutation and repair both require cellular proliferation [29].

Sonnenschein and Soto have suggested that proliferative activity, rather than quiescence, is the default condition of cells [20]; thus the natural impulse of free-living cells to proliferate is, in a multicellular animal, actively regulated by aspects of the multicellular environment. It seems logical, therefore, to propose that the abnormal proliferative activity in a cancer requires that certain suppressor genes, especially those that normally, in a post-embryonic animal, suppress embryonic development and/or wound healing, be silenced. My view of carcinogenesis depends, as I have said, upon the assumption that DNA damage in untranscribed genes is not repaired [28]. Therefore, it seems reasonable to assume that, over time in a tumor, mutations might tend to accumulate in any silenced gene or in non-coding segments of a gene.

The picture that emerges from these considerations is that neoplasia is probably caused by the reexpression of proliferation-enhancing development or wound-healing genes that, outside of a neoplasm, would usually be expressed only in embryonic life or during wound healing. Such reexpression, indeed overexpression, now as oncogenes, could be caused by either interference with communication between the oncogene and the appropriate suppressor gene or by the actual silencing of the suppressor gene by either mutation or, perhaps more commonly, by epigenetic influences. Mutations that may have occurred in the development genes might often be repaired, if and when these genes were reexpressed as oncogenes within a proliferating neoplasm. Since the oncogene's function is to drive the cancer, any mutations in these genes would probably be highly selected to retain the gene's normal or near normal function; there might thus be selection to produce only normal or near normal product, a product that might then be only minimally, if at all, immunogenic. Alternatively, any mutations that did occur among the oncogenes might not produce immunogenicity if the products of such mutations were not found on the cell surface.

## Recapitulation

Thus, my thesis is that *most *of the mutations actually found in a neoplasm would probably not be among the reexpressed proliferation-stimulating oncogenes, but would be among those suppressor genes that were newly silenced either during or after transformation and that remained silenced, unexpressed, and unrepaired throughout the life of the tumor. Because these suppressor genes had been silenced, their mutations would probably not result in new antigens.

To recapitulate, an important corollary of the previous discussion is the conclusion that the mutations that are identified by their associated antigens, are seldom cancer-inducing, but are random and incidental. The genes that actually drive the neoplasm, the so-called oncogenes, are embryonic development or wound-healing genes that are reexpressed and overexpressed in a tumor. Being highly selected in the tumor for essentially normal functions, the reexpression of these genes, even when mutated, might not produce new antigens, especially if the oncogene products did not appear on the cell surface. Mutations that occur in silenced suppressor genes go unrepaired, but because silent unexpressed genes have no product, these mutations also should fail to give rise to antigens. Thus, my view of carcinogenesis suggests that there are usually, with few exceptions, no mutations in the carcinogenic process that can produce new antigens unless a virus or an unrealistically high concentration of oncogen is involved. Mutations, induced by high concentrations of a chemical carcinogen do give rise to immunogenicity and thus they presumably occur in expressed genes, but, as already stated, such mutations are apparently not directly related to etiology and are usually unrealistic laboratory constructs.

In sum, the dismal record of the attempts to utilize anti-tumor immunity in the clinic seems entirely consistent with the idea that cancer is a disease based most often upon the silencing of suppressor genes, either by mutation or by epigenetic means. The epigenetic pathways may be the more frequent. The postulated, as well as demonstrated, paucity of antigen-producing mutations in the expressed genes of most cancers, plus the lack of antigenicity that would result from mutations that might arise in the silenced suppressor genes, explains the basic paucity of tumor-specific immunogenicity. Furthermore, the little tumor immunogenicity that may exist usually produces a reaction that is stimulatory, not inhibitory, to tumor proliferation.

Although there is certainly a role for immune suppressor cells and other blocking factors [30], in this essay I have suggested that the major difficulty lies in the fact that most cancers have little immunogenicity because of a basic lack of tumor-specific antigenicity. What weak antigens a tumor may have are usually tumor specific rather than tumor-type specific. The tissue-specific antigens involved in autoimmune reactions and the "carcino-embryonic antigens" are important realities, but have been difficult to utilize to any great extent in cancer prevention or therapy, probably in large part because of the powerful natural tolerance to such antigens. Perhaps further research will reveal ways to use the autoimmune mechanism against cancers that arise in disposible organs such as the prostate or the mammary gland where tumor specificity might be less important and organ specificity might be sufficient.

If my view of the carcinogenic process is correct, the utility of immune mechanisms in the "war on cancer" seems likely to remain, dispite some minor triumphs, rather dismal. However, the success of intravesicular BCG to treat carcinoma of the bladder offers hope [31]. There have also been encouraging reports about the probable immunogenicity of cutaneous melanoma [32,33]. Dispite my previously expressed view that immunosurveillance of cancer is unlikely, there is one observation that deserves special mention. It has long been known that cancer occurs with greatly increased frequency in patients who undergo prolonged immunodepression to facilitate kidney transplantation. The increased incidence is not general, but is largely confined to tumors of the lymphoid system or of the skin [34]. The excess of lymphoid tumors could be easily considered the ultimate consequence of compensatory hyperplasia in the damaged immunological organ, but the excess of skin tumors is more likely to have a direct immunological basis, either decreased surveillance or increased immunostimulation by the impaired immune mechanism. In either case, the fact that the excess tumor incidence is largely confined to the skin suggests that skin tumors may be unusually antigenic and/or the skin is an unusally active immunological organ.

My view of the very complex interaction of cancer and the immune reaction is certainly simplistic and perhaps overly pessimistic, but it may, I am afraid, capture the essence of the problem.
